# Locally Advanced, Unresectable Squamous Cell Carcinoma of the Gallbladder

**DOI:** 10.1155/2015/424650

**Published:** 2015-07-06

**Authors:** Timothy J. Weatherall, Moon Fenton, Gitonga Munene, Paxton V. Dickson, Jeremiah L. Deneve

**Affiliations:** ^1^Department of Surgery, University of Tennessee Health Sciences Center, 910 Madison Avenue, Suite 220, Memphis, TN 38163, USA; ^2^Department of Hematology/Oncology, The West Cancer Center, Memphis, TN, USA

## Abstract

Primary squamous cell carcinoma (SCC) of the gallbladder is a rare malignancy of the gallbladder, accounting for less than 5% of gallbladder pathology. Initial presentation is often similar to adenocarcinoma of the gallbladder. SCC tends to be more locally aggressive, however, and possesses a worse prognosis than adenocarcinoma. We report a case of locally advanced SCC of the gallbladder.

## 1. Case Report

**Figure 1:**
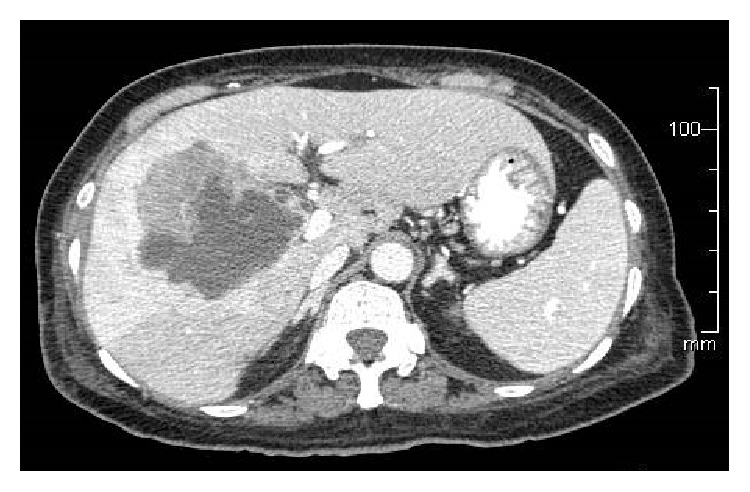
An 8.6 cm right-sided liver mass with associated abscess.

**Figure 2:**
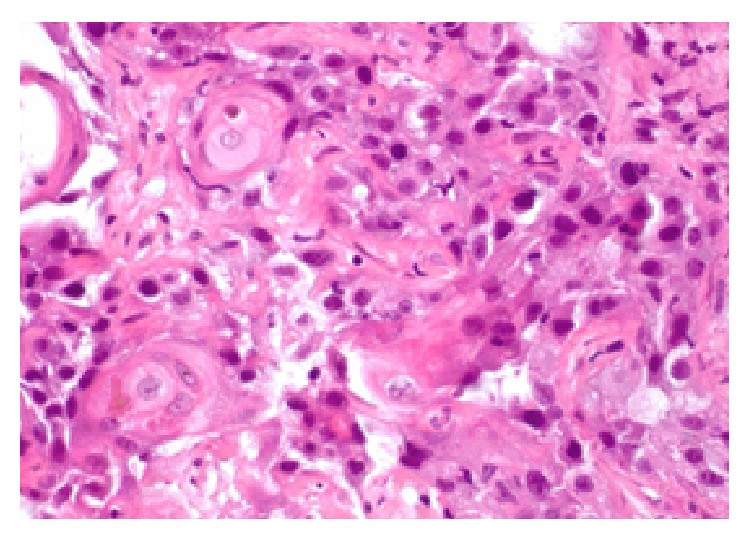
Percutaneous biopsy demonstrated squamous cell carcinoma histology.

**Figure 3:**
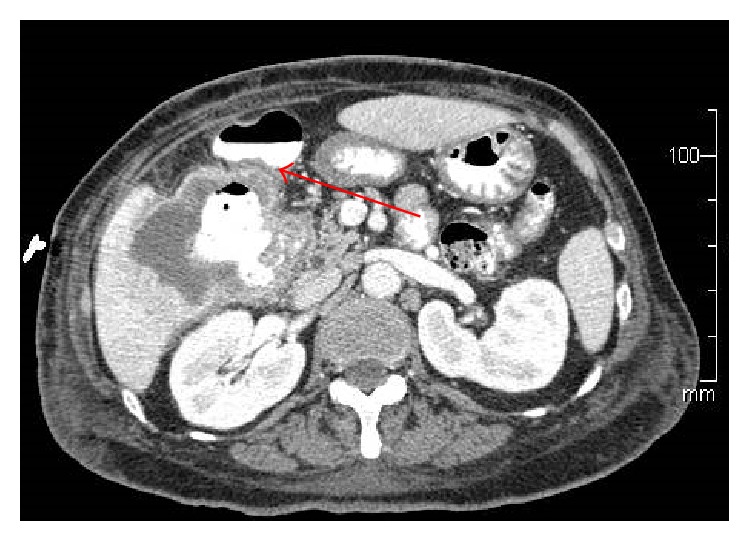
SCC of the gallbladder with fistula to the hepatic flexure of the colon.

**Figure 4:**
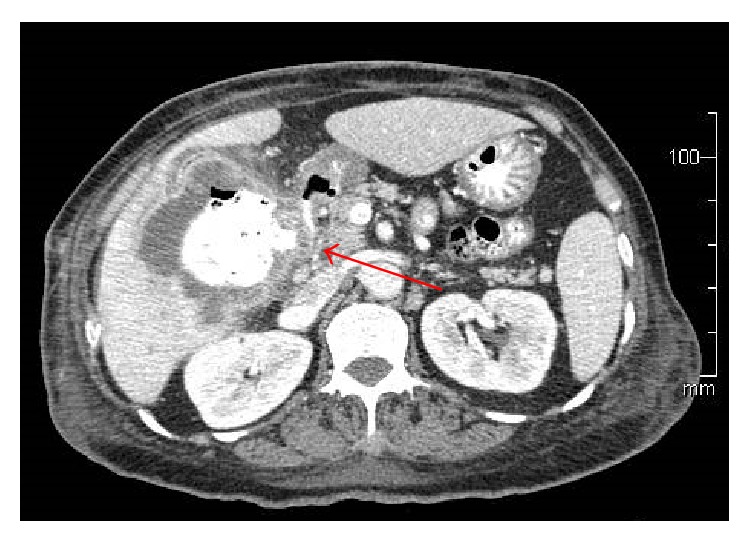
SCC of the gallbladder with fistula to the duodenum.

A 67-year-old Asian female presented to her primary care physician in March 2012 with persistent fever and chills with associated weakness and weight loss. She had no abdominal symptoms or history of gallbladder pathology. She was treated for bronchitis but her fever and chills persisted despite multiple rounds of oral antibiotic therapy. Further workup revealed a leukocytosis of 30,000 wbc/*μ*L. She underwent hematology/oncology evaluation to exclude a possible bone marrow dysplastic process. This did not demonstrate an underlying cause for the leukocytosis. She presented to a local emergency room in mid-June 2012 with complaints of chills, weakness, and malaise. She was admitted with Gram-negative rod bacteremia. A computed tomography (CT) scan was obtained which demonstrated an 8.6 cm mass of the right lobe of the liver with associated abscess (Figure 1). Biopsy of the lesion revealed SCC of the gallbladder (Figure 2). Percutaneous drainage of the abscess was performed which was significant for* E. coli* and* Enterococcus*. She was discharged on antibiotic therapy but readmitted ten days later with fever, chills, and failure to thrive. Repeat CT scan imaging revealed an increase in the size of the mass to 10.2 cm. Evidence of fistulization of the lesion to the gastric antrum, duodenum, and hepatic flexure of the colon was also noted (Figures 3 and 4). There was no distant metastasis or lymphadenopathy observed. She was referred to our institution in August 2012 for consideration of additional therapy. Magnetic resonance imaging revealed her tumor to be potentially resectable. However, because of her limited reserve and declining functional status, it was felt that she may not tolerate radical en bloc resection. Palliative chemotherapy was offered but declined. Comfort care measures were recommended and the patient passed away soon thereafter.

## 2. Discussion

Adenocarcinoma of the gallbladder is the most common malignancy of the gallbladder, accounting for up to 95% of cases [1]. While squamous differentiation associated with adenocarcinoma comprises a majority of the remainder of cases, pure primary SCC of the gallbladder is rare, accounting for less than 1% of gallbladder malignancies [2]. Microscopically, these tumors are characterized by substantial keratinization with abundant keratohyalin pearls and central deposition of dense keratin material within infiltrative nests (Figure 2). There have been associations of the development of SCC of the gallbladder with gallstones and/or parasitic infections, although the exact etiology is unknown [3].

The clinical presentation of SCC of the gallbladder is similar to other gallbladder pathologies. Women are more commonly affected than men with a peak incidence in the 6th decade [2]. Some patients may present with right upper quadrant pain or jaundice or are diagnosed incidentally after undergoing cholecystectomy for presumed cholecystitis. A majority of patients, however, present with large, bulky tumors that have often invaded adjacent organs [4]. Given the large tumor size and adjacent organ involvement, many patients are not considered surgical candidates at initial presentation. This may explain why patients with SCC of the gallbladder tend to behave more poorly than those with adenocarcinoma. Despite the locally aggressive nature, lymph node metastasis and/or distant metastases are rarely observed with SCC of the gallbladder.

Surgical resection is the primary treatment for SCC of the gallbladder with complete resection associated with improved survival [5]. When suspected preoperatively, the gallbladder with an adjacent rim of liver tissue should be resected. Lymphadenectomy can be performed if there is clinical suspicion of lymph node involvement. Even in the setting of adjacent organ involvement, surgical resection remains warranted. Kalayarasan and colleagues demonstrated that, despite larger tumor size and higher likelihood of adjacent organ involvement for patients with SCC of the gallbladder, complete resection is possible and is associated with similar survival when compared to patients with adenocarcinoma [4].

For those deemed unresectable or unable to undergo complete resection, the outcome is poor. Those with SCC of the gallbladder appear to have an even worse outcome than those with adenocarcinoma. Roa et al. reviewed 34 cases of squamous and adenosquamous carcinomas of the gallbladder [2]. The survival for SCC/adenosquamous was significantly worse than for adenocarcinoma. The median overall survival (OS) was 5 months, although a few long-term survivors were observed. Oohashi and colleagues noted that resection is warranted only if complete resection is possible [5]. Twenty-nine patients with adenosquamous/SCC of the gallbladder underwent resection. The estimated 5-year OS was 63% if complete resection (R0) was performed compared to 0% for those undergoing incomplete resection (R0/R1, *P* < 0.001).

In conclusion, SCC of the gallbladder is a rare disease process associated with a poor overall outcome. Patients often present with large, bulky tumors with involvement of adjacent organs. Lymph node involvement and distant metastasis are rare. Surgery is the mainstay of therapy and offers the only possibility for long-term survival. Unfortunately, many patients present with advanced stage disease, such as the one described, with limited treatment options.
